# A new *Hyphessobrycon* (Characiformes: Acestrorhamphidae) of the *Hyphessobrycon agulha* lineage of Hyphessobryconinae from the lower Aripuanã basin, Brazil, with comments about the lineage

**DOI:** 10.1111/jfb.70379

**Published:** 2026-02-26

**Authors:** Tiago C. Faria, Willian M. Ohara, Iann Leonardo Pinheiro Monteiro, Claudio Oliveira

**Affiliations:** ^1^ Laboratório de Biologia e Genética de Peixes (LBP), Departamento de Biologia Estrutural e Funcional, Instituto de Biociências de Botucatu (IBB) Universidade Estadual Paulista ‘Júlio de Mesquita Filho’ (UNESP) Botucatu Brazil; ^2^ Departamento de Biologia Instituto de Ciências Biológicas (ICB), Universidade Federal do Amazonas (UFAM) Manaus Brazil; ^3^ Laboratório de Ictiologia, Coordenação de Zoologia Museu Paraense Emílio Goeldi Belém Brazil

**Keywords:** biodiversity, *Hyphessobrycon ericae*, *Hyphessobrycon ribeiroi*, ichthyology, Rio Amazonas

## Abstract

A new species of *Hyphessobrycon* is described from a tributary of Rio Jatuarana, lower Rio Aripuanã basin, Rio Madeira basin, Apuí, Amazonas. The new species is part of the *Hyphessobrycon agulha* lineage, with the typical midlateral narrow black stripe immediately followed dorsally by an iridescent stripe. Its phylogenetic position is corroborated by the DNA barcoding methodology, which also indicates the new species as closely related to *Hyphessobrycon ericae* and *Hyphessobrycon ribeiroi*, with both possessing very distinct colour patterns. The new species can be distinguished from all species of *Hyphessobrycon* by the association of a well‐defined and horizontally elongated humeral blotch with a ventral diffuse expansion, a conspicuous caudal peduncle blotch restricted to the ventral half of the caudal peduncle and proximal half of mid rays of caudal fin, the presence of a red midlateral stripe dorsal to the iridescent stripe and lateral‐line scale counts.

## 1 INTRODUCTION

*Hyphessobrycon* Durbin 1908 is the largest genus of Acestrorhamphidae, with 148 species currently recognized as valid (Lima et al., 2025; Toledo‐Piza et al., 2024). The large number of species is directly related to its non‐monophyletic status, as recovered by morphological and molecular phylogenies (Melo et al., 2024; Mirande, 2018). Putative monophyletic groups have been proposed among species of the genus to allow the possibility to work with subgroups among its numerous species, with most of these groups being proposed using, mainly, colour pattern characters (Ingenito et al., 2013; Lima et al., 2014; Ohara & Lima, 2015). The *Hyphessobrycon heterorhabdus* species group (Lima et al., 2014; Moreira & Lima, 2017; Faria, Bastos, et al., 2020) and the *Hyphessobrycon agulha* species group (Faria, Lima, & Wosiacki, 2020; Ohara & Lima, 2015) are especially relevant in the context of the present species description.

**FIGURE 1:**
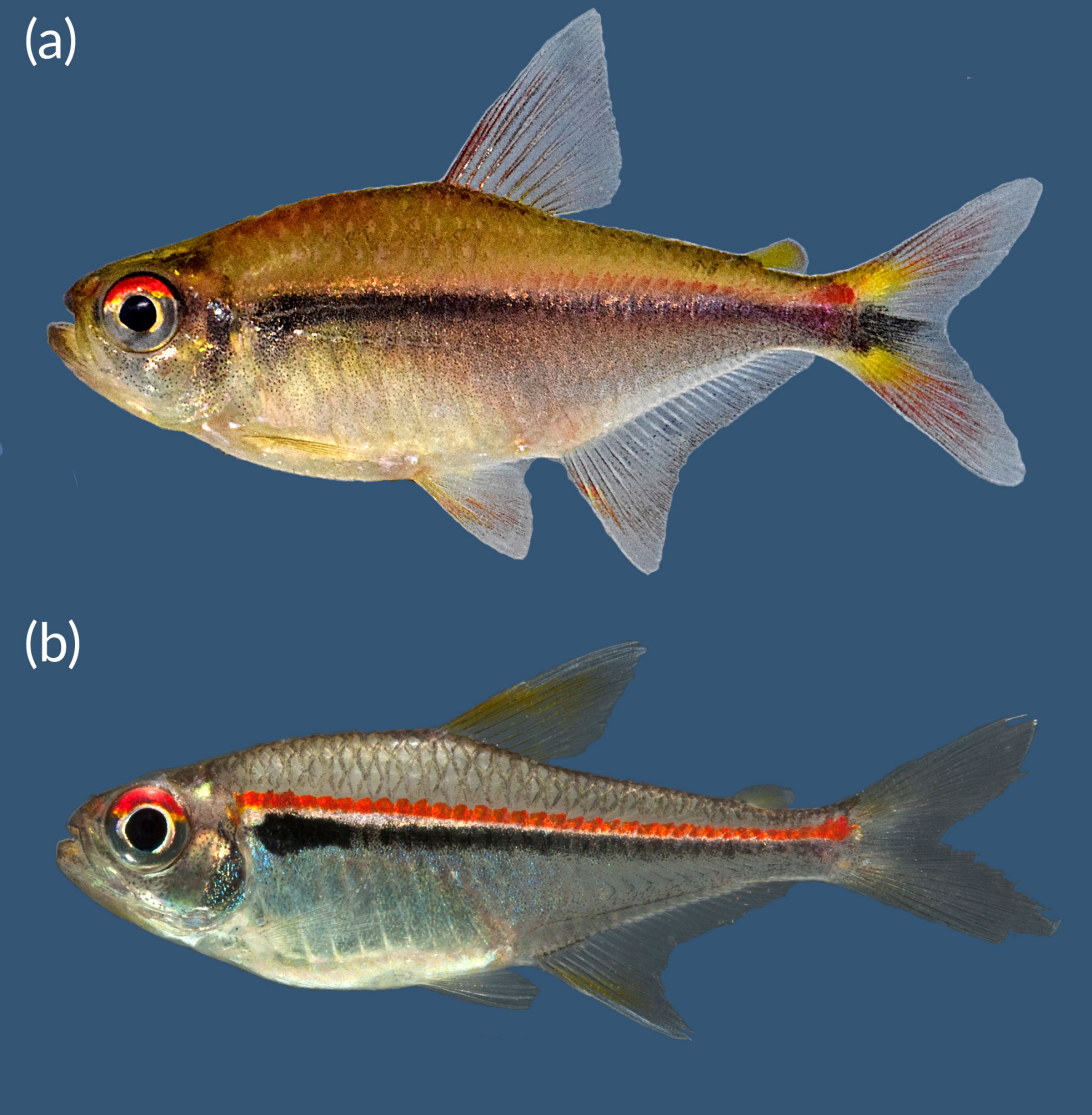
*Hyphessobrycon heterorhabdus* and *Hyphessobrycon agulha* species groups representatives. (a) *Hy. heterorhabdus*, ZUEC 17135, female, Brazil, Belém region, picture of Douglas A. Bastos. (b) *Hy. agulha*, LBP 33136, female, Brazil, Rio Mamuru basin.

The *Hy. agulha* species group (see Figure 1a), with *Hy. agulha* Fowler, 1913, *Hyphessobrycon clavatus* Zarske, 2015, *Hyphessobrycon eschwartzae* García‐Alzate, Román‐Valencia and Ortega, 2013, *Hyphessobrycon herbertaxelrodi* Géry, 1961, *Hyphessobrycon klausanni* Garcia‐Alzate, Urbano‐Bonilla & Taphorn, 2017, *Hyphessobrycon loretoensis* Ladiges, 1938, *Hyphessobrycon lucenorum* Ohara & Lima, 2015, *Hyphessobrycon margitae* Zarske, 2016, *Hyphessobrycon metae* Eigenmann & Henn, 1914, *Hyphessobrycon mutabilis* Costa & Géry, 1994, *Hyphessobrycon peruvianus* Ladiges, 1938, *Hyphessobrycon wadai* Marinho, Dagosta and Oyakawa, 2016, and *Hyphessobrycon zoe* Faria, Lima, & Wosiacki, 2020, is diagnosed by the presence of a broad midlateral diffuse stripe that, many times, occupies the ventral half of the body, especially on the caudal peduncle (Faria, Lima, & Wosiacki, 2020; Géry, 1977; Ohara & Lima, 2015).

The *Hy. heterorhabdus* species group (see Figure 1b), comprises *Hyphessobrycon amapaensis* Zarske, Géry, 1998; *Hy. heterorhabdus* (Ulrey, 1894); *Hyphessobrycon ericae* Moreira & Lima, 2017; *Hyphessobrycon montagi* Lima et al., 2014; *Hyphessobrycon sateremawe* Faria, Lima, & Wosiacki, 2020, Faria, Bastos, et al., 2020, and *Hyphessobrycon wosiackii* Moreira & Lima, 2017, and characterized by a tricoloured longitudinal pattern, composed dorsally by a red or reddish longitudinal stripe, a middle iridescent, golden or silvery longitudinal stripe, together with the more ventrally developed longitudinal dark pattern consisting of a humeral blotch and a dark midlateral stripe. The humeral blotch is horizontally elongated, with a posterior region more diffuse that merges with a midlateral stripe that becomes progressively more diffuse towards the caudal peduncle (Faria et al., 2021; Faria, Bastos, et al., 2020; Lima et al., 2014; Moreira & Lima, 2017).

Hyphessobryconinae, one of the new Acestrorhamphidae subfamilies recently proposed using phylogenomic data (Melo et al., 2024), is composed of four main clades, one of which was informally named as *Hy. agulha* lineage (Faria et al., 2025), comprises all sampled species previously included in the *Hy. agulha* and *Hy. heterorhabdus* species groups, with the sole exception of *Hy. wadai*, which was recovered in the sister clade. The *Hy. agulha* lineage includes *Hy. agulha*, *Hy. amapaensis*, *Hy. ericae*, *Hy. herbertaxelrodi*, *Hy. heterorhabdus*, *Hy. margitae*, *Hy. mutabilis*, *Hy. peruvianus*, *Hemigrammus bellottii* (Steindachner, 1882) and *Hemigrammus rubrostriatus* Zarske, 2015, the last two not included in the aforementioned putative groups, likely by being part of *Hemigrammus*. In the present study, a new species of *Hyphessobrycon* of the *Hy*. *agulha* lineage of Hyphessobryconinae from the lower Aripuanã basin, Brazil, is described, and we present comments about species of the lineage.

## 2 MATERIALS AND METHODS

Specimens used in the present study were collected using the ICMBIO licence number 13843–5 according to Brazilian law. Institutional abbreviations follow Sabaj (2020, 2025).

### 2.1 Measurements and counts

Counts and measurements follow Fink and Weitzman (1974), except for the number of horizontal scale rows below the lateral line, which are counted to the pelvic‐fin insertion (excluding the axillary scale) rather than to the anal‐fin origin, and the addition of two measurements: dorsal‐fin base length and anal‐fin base length. Standard length (SL) is expressed in millimetres, and all other measurements are expressed as percentages of SL, except subunits of the head, which are expressed as percentages of head length (HL). In the description, counts are followed by their absolute frequency in parentheses. Asterisks indicate the counts of the holotype. *Circuli* and *radii* were counted on scales from the row immediately dorsal to the lateral line at the vertical through the dorsal‐fin origin. Counts of supraneurals, branchiostegal rays, gill rakers of the first branchial arch, teeth, teeth cusps, unbranched anal‐fin rays, procurrent caudal‐fin rays and position of pterygiophores were taken from cleared and stained (C&S) specimens prepared according to Taylor and Van Dyke (1985). Vertebrae of the Weberian apparatus were counted as four elements and the compound caudal centrum (PU1 + U1) as a single element. Catalogue numbers are followed by the total number of specimens and their SL range. The number of cleared and stained specimens (indicated by C&S) is given in parentheses, followed by their respective SL range.

### 2.2 Molecular analysis

DNA extraction followed Ivanova et al. (2006), and partial sequences of the mitochondrial gene cytochrome c oxidase subunit I (COI) were amplified by polymerase chain reaction (PCR), with primers FishF1/R1 described by Ward et al. (2005). Reactions were carried out in a 12.5 μL reaction volume containing 1.25 μL of 10× PCR buffer, 0.40 μL MgCl_2_ (50 mM), 0.30 μL dNTPs (2 mM), 0.25 μL of each primer (5 μM), 0.20 μL of PHT Taq DNA polymerase (Phoneutria), 2 μL DNA template (200 ng) and 7.85 μL of ddH_2_O. The PCR consisted of denaturation (5 min at 95°C) followed by 30 cycles of denaturation (1 min at 95°C), primer hybridization (45 s at 52°C), nucleotide extension (1 min at 68°C) and a final extension (10 min at 68°C). All PCR products were checked using 1% agarose gel and purified with ExoSap‐IT (USB Corporation) following the manufacturer's instructions. The purified PCR products were sequenced using the Big DyeTM Terminator version 3.1 Cycle Sequencing Ready Reaction Kit (Applied Biosystems, Austins, USA) and purified through ethanol precipitation. Amplified fragments were then loaded into an ABI 3500 Genetic Analyser (Applied Biosystems), in the Instituto de Biotecnologia (IBTEC), Instituto de Biociências, Universidade Estadual Paulista Júlio de Mesquita Filho, Botucatu, Brazil. For this study, we generated two sequences of the new species and 24 sequences of other 16 species of *Hyphessobrycon*, *Hemigrammus*, *Erythrocharax*, *Jupiaba* and *Tucanoichthys*, including species of Hyphessobryconinae, Tyttobryconinae and *Hy. pulchripinnis* as out‐group. We also used two sequences of *Hy. heterorhabdus*, one sequence of *Hy. loretoensis* and one sequence of *Hyphessobrycon nigricinctus* obtained from GenBank. For more details about sequences and GenBank numbers, see Table 1. The sequences were assembled using the software Geneious 7.1.4 (Kearse et al., 2012) and aligned with Muscle (Edgar, 2004) under default parameters. The best‐fit model of nucleotide evolution was selected based on Akaike Information Criterion with corrections for small sample sizes (AICc). The overall mean genetic distances (among all specimens), as well as interspecific (among species group) and intraspecific distances (among specimens of each species group), were estimated with 1.000 pseudoreplicates and without root. These previous analyses were estimated using MEGA version 11 (Tamura et al., 2021). Maximum likelihood (ML) analysis was performed in RAxML‐HPC version 8 on ACCESS using the GTRGAMMA model in the CIPRES server. The best tree was accessed through 10 random searches with 1000 bootstrap pseudoreplicates. The resulting ML tree was used as an input tree for the Poisson Tree Process (PTP) model analysis (Zhang et al., 2013), which was performed on the PTP web server (https://species.h-its.org), with the option ‘remove outgroup’ and the other parameters in default. The analysis of Assemble Species by Automatic Partitioning (ASAP) (ASAP; Puillandre et al., 2021) is available in the ASAP webserver (https://bioinfo.mnhn.fr/abi/ public/asap/asapweb.html) with model Jukes‐Cantor (JC69).

**TABLE 1:** Information on DNA barcoding sequence vouchers and GenBank/BOLD codes.

Species	Collection	Voucher	River basin	Specific location	Municipality	Co‐ordinates	BOLD	GenBank
*Hyphessobrycon pastanai*	LBP 32949	LBP 112479	Rio Jatuarana, Aripuanã, Madeira	Igarapé afl. Rio Jatuarana	Apuí(AM)	07°26′25.94″ S 60°26′27.46″ W		PP786523
*Hy. pastanai*	LBP 32949	LBP 112480	Rio Jatuarana, Aripuanã, Madeira	Igarapé afl. Rio Jatuarana	Apuí(AM)	07°26′25.94″ S 60°26′27.46″ W		PP786524
*Hyphessobrycon montage*	LBP 31642	LBP 110260	Rio Arapiuns, Tapajós	Aquarium specimen				PP786520
*Hyphessobrycon mamuruensis*	LBP 33015	LBP 112842	Rio Mamuru	Igarapé afl. Rio Mamuru	Itaituba (PA)	04°04′46.03″ S 56°11′45.41″ W		PP786521
*Hy. mamuruensis*	LBP 33015	LBP 112841	Rio Mamuru	Igarapé afl. Rio Mamuru	(Itaituba (PA)	04°04′46.03″ S 56°11′45.41″ W		PP786522
*Hyphessobrycon ribeiroi*	LBP 31614	LBP 110179	Rio Tapajós	Igarapé Sonrisal	Santarém (PA)	02°32′02.2”S, 54°55′23.9”W		PP372842
*Hy. ribeiroi*	LBP 31614	LBP 110180	Rio Tapajós	Igarapé Sonrisal	Santarém (PA)	02°32′02.2”S, 54°55′23.9”W		PP372843
*Hyphessobrycon* cf. *ericae*	LBP 32946	LBP 112470	Rio Sucunduri	Rio Camaiú	Maués (AM)	06°55′59.81″ S, 59°19′44.65″ W		PP372831
*Hy*. cf. *ericae*	LBP 31628	LBP 110207	Rio Curuá‐Una	Rio Moju	Belterra (PA)	03°25′05.6” S, 54°54′46.50” W		PP372834
*Hy*. cf. *ericae*	LBP 31628	LBP 110208	Rio Curuá‐Una	Rio Moju	Belterra (PA)	03°25′05.6” S, 54°54′46.50” W		PP372835
*Hy*. cf. *ericae*	LBP 12107	LBP 51771	Rio Madeira	Rio Quinta	Porto Velho(RO)	09°03′40.6” S, 64°01′16.3” W		PP372836
*Hy*. cf. *ericae*	LBP 12107	LBP 51772	Rio Madeira	Rio Quinta	Porto Velho(RO)	09°03′40.6” S, 64°01′16.3” W		PP372837
*Hyphessobrycon herbertaxelrodi*	LBP 8387	LBP 40453	Rio Paraguai	Córrego Águas Claras	Tangará da Serra(MT)	S 14°21′03.2′, W 57°33′07.2”		PP372838
*Hyphessobrycon amapaensis*	LBP 30508	LBP 116901	Rio Amazonas	Igarapé afl. Rio Pedreira	Ferreira Gomes (AP)	00°41′44.0” N, 51°21′39.0” W		PP372830
*Hyphessobrycon heterorhabdus*	LBP 9451	LBP 45139	Rio Guamá	Igarapé afl. Igarapé São José	Santa Isabel (PA)	01°16′54.1” S, 48°10′09.9” W	ACD9730	
*Hyphessobrycon agulha*	LBP 34966 (from UFRO 5459)	LBP 116904, 6952.3 (UFRO)	Rio Madeira	Igarapé afl. Rio Aponiã	Porto Velho(RO)	08°04′43,3” S, 63°28′36” W		PP372829
*Hyphessobrycon loretoensis*		IIAP‐CIIAP‐01076‐3						MH411298‐1
*Hyphessobrycon nigricinctus*		IIAP‐CIIAP‐01162‐2						MK861658‐1
*Hyphessobrycon sateremawe*	INPA 50729	P 30449	Rio Abacaxis	Igarapé afl. rio Abacaxis	Nova Olinda do Norte(AM)	04°17′5” S, 58°34′44” W		PQ044051
*Hyphessobrycon eschwartzae*	ROMCID 134066 tissue	ROMI T10194M	Rio Madre de Dios	Rio Planchon	Tambopata (Madre de Dios, Peru)*	12°16′37.67” S, 69° 9′8.53” W		PQ044050
*Hyphessobrycon cantoi*	LBP 110138	LBP 31598	Rio Amazonas	Igarapé do Diamantino	Santarém (PA)	2°30′16.26” S, 54°39′33.12” W		PX441627
*Erythrocharax altipinnis*	LBP 69052	LBP 10881	Rio Tapajós	Rio Curuá	Altamira (PA)	08°53′54” S, 54°59′20” W		PX441628
*Hemigrammus levis*	LBP 83469	LBP 21027	Rio Amazonas	Rio Calçoene	Calçoene (AP)	02°31′08.9” N, 51°00′52.9” W		PX441629
*Jupiaba keithi*	LBP 82852	LBP 21122	Rio Oiapoque	Igarapé do Quatorze	Oiapoque (AP)	03°45′10.4” N, 51°46′57.3” W		PX441630
*Hyphessobrycon pulchripinnis*	LBP 32962	LBP 112719	Rio Tapajós	Igarapé do Abacaxi	Jacareacanga (PA)	06°08′51.9″ S, 57°43′44.3″ W		PP372832
*Tucanoichthys tucano*	LBP 18388	LBP 74648	Aquarium material		Brazil			PX441631

## 3 RESULTS

*Hyphessobrycon pastanai*, new species

urn:lsid:zoobank.org:pub:47CACABA‐37BC‐4CB5‐9D6E‐B47263B6D052

urn:lsid:zoobank.org:act:36B6B9E1‐7BBB‐413C‐96BF‐9B3CF703C978

Order: Characiformes Goodrich, 1909.

Family: Acestrorhamphidae Eigenmann, 1907.

**FIGURE 2:**
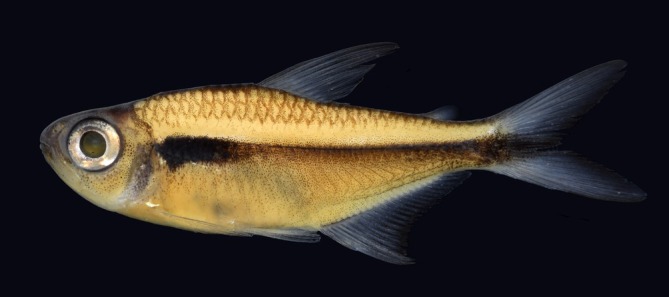
*Hyphessobrycon pastanai* new species. LBP 34081, holotype, 29.4 mm standard length (SL), male, Brazil, Amazonas, Apuí, stream tributary of Rio Jatuarana, Rio Aripuanã basin, Rio Madeira basin.

**FIGURE 3:**
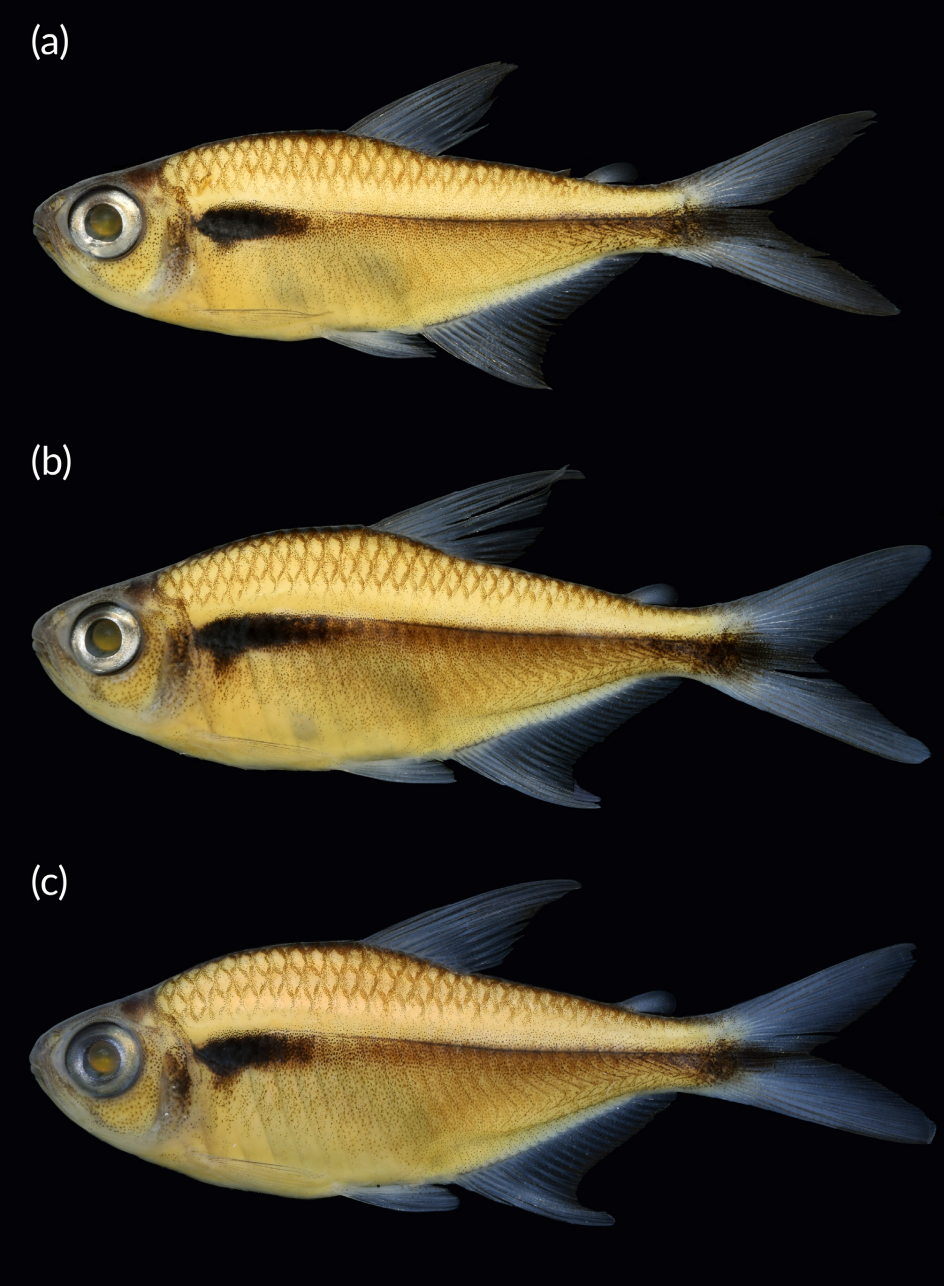
Paratypes, LBP 32949, same locality of holotype. (a) Paratype, 29.4 mm standard length (SL), male. (b) Paratype, 31.9 mm SL, female. (c) Paratype, 31.4 mm SL, female.

**FIGURE 4:**
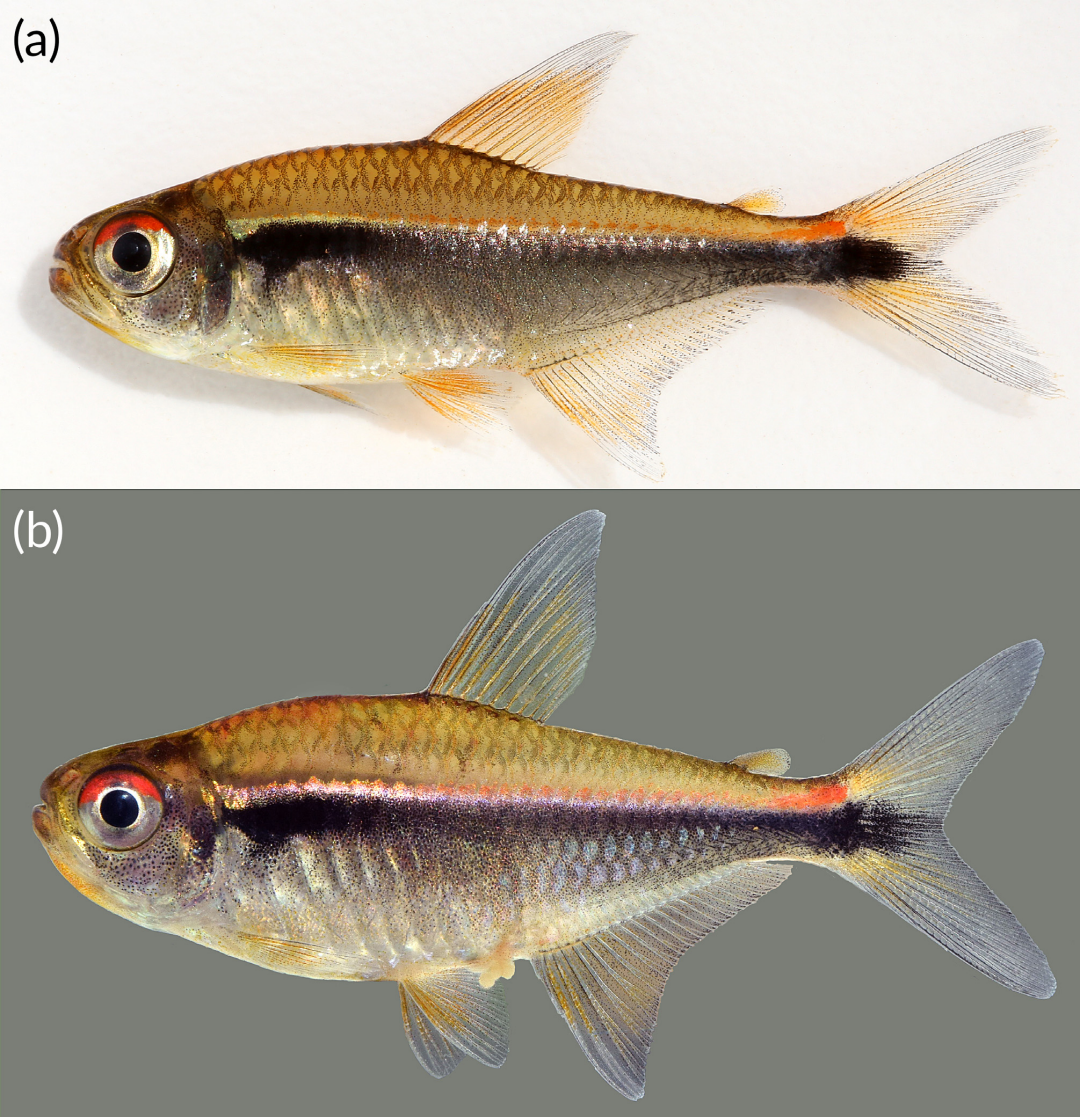
*Hyphessobrycon pastanai* n. sp. Living specimens. (a) MZUSP 117609, unsexed, same locality as holotype. (b) LBP 32949, female, same locality as holotype.

Genus: *Hyphessobrycon* Durbin, 1908.

(Figures 2, 3, 4; Table 2).

**TABLE 2:** Morphometric data for *Hyphessobrycon pastanai*.

	Holotype	Range	Mean ± SD	*N*
Standard length(mm)	29.4	27.0–31.9	–	
Percentages of standard length				
Depth at dorsal‐fin origin	30.9	28.0–34.1	31.0 ± 1.5	30
Snout to dorsal‐fin origin	48.0	47.2–50.2	48.8 ± 0.7	30
Snout to pelvic‐fin origin	46.6	44.4–48.0	46.1 ± 0.8	30
Snout to anal‐fin origin	62.6	58.7–65.8	61.4 ± 1.4	30
Caudal peduncle depth	8.5	7.7–9.5	8.4 ± 0.4	30
Caudal peduncle length	12.6	11.4–14.5	12.8 ± 0.8	30
Pectoral‐fin length	22.1	19.0–22.3	20.4 ± 0.9	30
Pelvic‐fin length	16.0	14.3–17.5	16.2 ± 0.7	30
Dorsal‐fin base	12.9	12.1–14.8	13.2 ± 0.6	30
Dorsal‐fin length	31.6	28.2–31.6	30.2 ± 0.9	30
Anal‐fin base	28.9	26.3–31.1	27.9 ± 1.0	30
Head length	26.9	24.9–27.2	26.1 ± 0.6	30
Percentages of head length				
Horizontal orbital diameter	40.5	34.1–41.2	38.1 ± 1.6	30
Snout length	20.5	18.9–25.6	22.2 ± 1.7	30
Least interorbital width	27.8	27.1–31.7	28.9 ± 1.2	30
Upper‐jaw length	44.3	41.3–47.6	43.6 ± 1.5	30

*Note*: *N* (number of specimens measured) = 30.

Abbreviation: SD, standard deviation.

### 3.1 Holotype

LBP 34081, 29.4 mm SL, Brazil, Amazonas State, Apuí, stream tributary of Igarapé do Tamanduá, Rio Jatuarana, lower Rio Aripuanã basin, Rio Madeira basin, 07°26′25.94″ S 60°26′27.46″ W, T.C. Faria, I.L.P. Monteiro and M.A. Pinheiro, 8 December 2022.

### 3.2 Paratypes

All from same locality as holotype. ROM 114796, 5, 27.1–30.1 mm SL, LBP 32949, 111, 5 CS, 15.5–31.9 mm SL, INPA 61185, 5, 29.2–31.5 mm SL, ZUEC 17662, 10, 21.5–31.5 mm SL, same data as holotype. MZUSP 117609, 8, 15.8–31.1 mm SL, W.M. Ohara and V. Abrahão, 22 June 2015.

### 3.3 Diagnosis

*Hyphessobrycon pastanai* can be distinguished from all congeners, except *Hy. agulha*, *Hy. amapaensis*, *Hyphessobrycon cantoi* Faria et al., 2021, *Hy. cyanotaenia* Zarske & Géry, 2006, *Hy. ericae*, *Hy. eschwartzae*, *Hy*. *heterorhabdus*, *Hy. herbertaxelrodi*, *Hy. sateremawe* and *Hyphessobrycon vilmae* Géry, 1966, by the presence of a midlateral dark stripe and by the presence in living specimens of a midlateral iridescent stripe (vs. the absence of one or both colour patterns described). *Hyphessobrycon pastanai* can be distinguished from *Hy. amapaensis*, *Hy. cantoi*, *Hy. cyanotaenia*, *Hy. eschwartzae*, *Hy*. *heterorhabdus*, *Hy. sateremawe* and *Hy. vilmae* by the presence of a horizontally elongated humeral blotch with conspicuous diffuse ventral expansion on the anterior region (vs. humeral blotch absent, indistinguishable from longitudinal broad dark stripe, or with no conspicuous ventral expansion). *Hyphessobrycon pastanai* can be distinguished from *Hy. agulha* by the presence of bony hooks exclusively on the pelvic fin of mature males (vs. the presence of bony hooks on the pelvic fin and anterior rays of the anal fin of mature males, or in all rayed fins of very developed males), by the presence of a conspicuous caudal peduncle blotch strongly marked (vs. caudal peduncle blotch weakly marked) and by the presence of 7 to 9 pored scales on the lateral line (vs. 10–35 pored scales on the lateral line, with the smallest number of a population ranging between 10 and 15). *Hyphessobrycon pastanai* can be distinguished from *Hy. herbertaxelrodi* by the presence of a caudal peduncle blotch (vs. caudal peduncle blotch absent) and by the presence, in life, of a red longitudinal stripe dorsal to the iridescent stripe that ends as a thickened red blotch on the caudal peduncle (vs. absence of red stripe). *Hyphessobrycon pastanai* can be distinguished from *Hy. ericae* by the presence of horizontally elongated caudal peduncle blotch that occupies most of the ventral half of the caudal peduncle, approximately above ventral procurrent rays, and the proximal half of the mid rays of the caudal fin (vs. presence of approximately elliptic caudal peduncle blotch that occupies most of the height of the caudal peduncle terminus and proximal half of mid rays).

### 3.4 Description

Morphometric data for holotype and paratypes are presented in Table 2. Body compressed. Greatest body depth at vertical through dorsal‐fin origin. Dorsal profile of head slightly convex from upper lip to vertical through posterior nostril, straight from that point to tip of supraoccipital spine. Dorsal profile of body slightly convex from latter point to anterior terminus of dorsal fin. Dorsal‐fin base straight, posteroventrally slanted, slightly convex from posterior terminus of dorsal‐fin to adipose‐fin insertion and slightly concave between adipose‐fin insertion and origin of anteriormost dorsal procurrent caudal‐fin ray. Ventral profile of head and body convex from tip of lower jaw to anal‐fin terminus. Ventral profile of caudal peduncle slightly concave.

Jaws equal, mouth terminal. Posterior terminus of maxilla reaching vertical through anterior margin of iris. Maxilla approximately at 45 degrees angle relative to longitudinal axis of body. Nostrils close to each other, anterior opening oval, posterior opening crescent‐shaped. Premaxillary teeth in two rows. Outer teeth row with 3(4), or 4(1) bi‐(1) or tricuspid(15) teeth. Inner row with 5(4) or 6(1) uni‐(smaller teeth in specimen with six teeth), tri‐, tetra‐ or pentacuspid teeth, symphyseal tooth narrower than adjacent teeth. Maxilla with 3(1), 4(3) or 5(1) conical to tricuspid teeth. Dentary with 12(1), 15(1), 16(1) or 17(1) teeth, anteriormost 4 teeth larger, tri‐ to tetracuspid, fifth tooth intermediary in size uni‐ or bicuspid (absent in one specimen), posterior teeth smaller, with small variation in size, and conical. Central cusp of all teeth more developed than remaining lateral cusps.

Scales cycloid. Three to six *radii* strongly marked, *circuli* well marked anteriorly, weakly marked posteriorly. Lateral line slightly deflected downward and incompletely pored, with 7(4), 8*(17) or 9(9) perforated scales. Longitudinal scales series including lateral‐line scales 33(1), 34*(10), 35(15) or 36(3). Longitudinal scale rows between dorsal‐fin origin and lateral line 5*(30). Longitudinal scale rows between lateral line and pelvic‐fin origin 3(12) or 4*(18). Predorsal scales 10*(18), 11(10) or 12(2). Circumpeduncular scales 12*(29). Caudal fin with few small scales basally.

Dorsal‐fin rays ii,8(1) or 9*(29). Dorsal‐fin origin slightly anterior from middle of standard length. First dorsal‐fin pterygiophore inserting behind neural spine of 9th(5) vertebrae. Adipose fin present. Anteriormost anal‐fin pterygiophore inserting posterior to haemal spine of 15th(3) or 16th(2) vertebrae. Anal‐fin rays iv,18(2), 19(7), 20*(12), 21(7) or 22(2). Last unbranched and first to third anteriormost branched rays distinctly longer than remaining rays, subsequent rays gradually decreasing in size. Pectoral‐fin rays i,10*(14), 11(15) or 12(1). Pelvic‐fin rays i,7*(30). Tip of pelvic fin reaching insertion of anteriormost anal‐fin rays. Caudal fin forked, lobes roughly rounded and of similar size. Nine (2), 10(2) or 13(1) dorsal procurrent caudal‐fin rays, and 7(1) or 8(4) ventral procurrent caudal‐fin rays. Vertebrae 34(5). Supraneurals 4(2) or 5(3), upper portion wider. Branchiostegal rays 4. First gill arch with 1(3) or 2(2) hypobranchial, 0(3) or 1(2) on cartilage between hypobranchial and ceratobranchial, 8(3) or 9(2) ceratobranchial, 1(5) on cartilage between ceratobranchial and epibranchial and 5(3) or 6(2) epibranchial gill rakers.

### 3.5 Colour in alcohol

Overall body colour beige. Middle portion of body darker. Dorsal portion of head dark. Ventralmost portion of head and body with low concentration of scattered dark chromatophores. Snout and dentary tip dark. Dark chromatophores abundant across infraorbitals and opercle. Predorsal and preadipose scales with conspicuous central dark blotches. Predorsal, preadipose and three dorsalmost scale rows with conspicuous reticulated pattern formed by dark chromatophores concentrated at scales margins. Reticulated pattern of dorsalmost scale rows darker. Area below reticulated pattern and immediately above humeral blotch and midlateral dark stripe clear, with scattered dark chromatophores. Humeral blotch horizontally elongated, anteriorly dark and well defined, progressively becoming diffuse posteriorly and ending around vertical through dorsal‐fin origin. Ventral expansion on the anteriormost region of humeral blotch, more diffuse than horizontal axis of humeral blotch, extending to the middle point between pectoral‐fin insertion and horizontal axis of humeral blotch. Posterior region of humeral blotch coalescing with midlateral narrow dark stripe. Midlateral dark stripe narrow and faint, extending from humeral blotch to caudal peduncle blotch. Narrow subjacent longitudinal dark line extending along horizontal septum, from end of humeral blotched to end of caudal peduncle. Ventral region of body darkened by many dark chromatophores widespread, less concentrated on abdominal region, nearly absent in ventralmost portion of abdominal region and more concentrated above anus, anal fin and caudal peduncle. Dark chromatophores aligned along myocommata of hypaxial muscles above anal fin and parallel to anal‐fin base. Caudal peduncle blotch diffuse, conspicuous, extending from end of midlateral thin dark stripe to mid rays of caudal fin. Caudal fin mostly hyaline where caudal peduncle blotch dark chromatophores are absent. Anal fin with dark chromatophores scattered along interradial membranes. Dorsal‐fin rays mostly hyaline, with dark chromatophores concentrated on anteriormost rays. Adipose fin with few scattered dark chromatophores. Pectoral fin hyaline. Pelvic fin with few dark chromatophores scattered mainly on distal region.

### 3.6 Colour in life

As shown in Figure 4, overall body colour is olive. Lower half of head and abdominal region silvery. Dorsal portion of eye red. Snout and ventral region of head orange. Tricolour longitudinal pattern extending from immediately posterior to opercle to base of caudal fin composed of a dorsal red stripe, a middle iridescent greenish stripe and a ventral dark longitudinal pattern. Red stripe narrow and interrupted, forming a row of red blotches but becoming continuous on caudal peduncle, where it becomes thicker and forms an horizontally elongated red blotch slightly more dorsal. Iridescent stripe continuous in all its extension and ending slightly before caudal peduncle red blotch. Dark longitudinal pattern composed of humeral blotch and midlateral dark thin stripe. Iridescent chromatophores scattered across ventral region of body and opercle. All proximal regions of fins (except anal fin) and anal fin (except anal‐fin lobe) orange.

### 3.7 Sexual dimorphism

Last unbranched and two anteriormost branched anal‐fin rays larger in females, resulting in a slightly more pointed and developed anal‐fin lobe (compare Figure 3a,b with Figures 3c and 2). Males present tiny bony hooks on all length of pelvic rays. Females reach larger sizes than males (largest female with 31.8 mm SL and largest male with 29.4 mm SL).

### 3.8 Distribution

**FIGURE 5:**
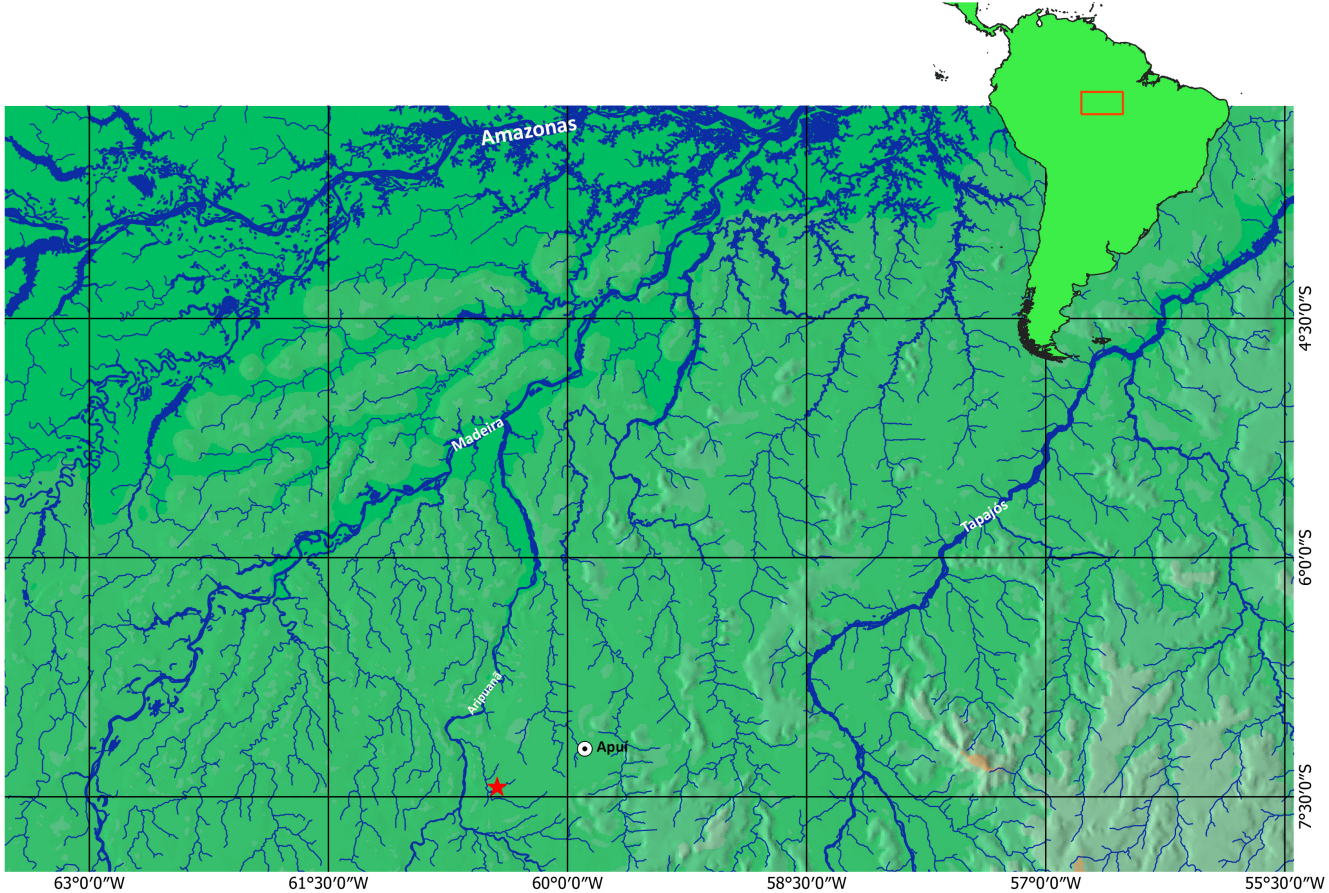
Map of the Rio Madeira region, showing the type locality of *Hyphessobrycon pastanai* (red star).

*Hyphessobrycon pastanai* is known only from its type locality, a small stream affluent of Rio Jatuarana, lower Rio Aripuanã basin, Rio Madeira basin, in Apuí city, state of Amazonas, Brazil (Figure 5).

### 3.9 Ecological notes

*Hyphessobrycon pastanai* is known from moderate‐flowing, slightly black water streams covered by forest vegetation and with rock bottom. Specimens were collected swimming on open water. Females with eggs were captured in December (Figure 4b). Syntopic species were *Bryconops* sp., *Moenkhausia oligolepis* (Günther 1864), *Saxatilia* sp. and *Moenkhausia* sp.

### 3.10 Etymology

The specific epithet is a homage to Dr. Murilo Nogueira de Lima Pastana, dear friend, ichthyologist and curator in the Museu de Zoologia da Universidade Estadual de São Paulo, as a recognition of its important contributions to the knowledge of fishes from the Apuí region. A genitive noun.

### 3.11 Genetics

**FIGURE 6:**
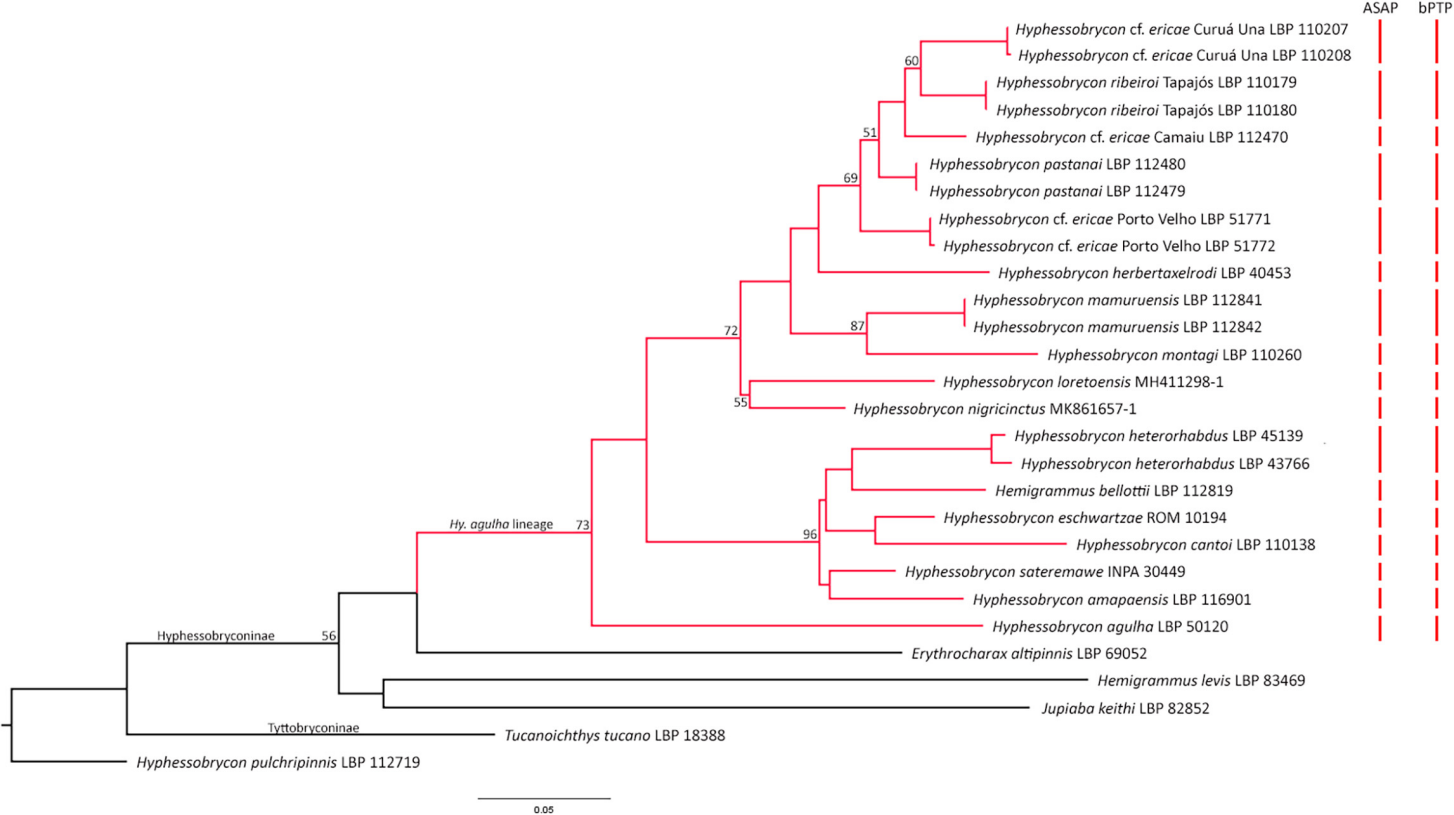
RaxML phylogenetic tree of *Hyphessobrycon pastanai* and the most similar species using cytochrome c oxidase subunit I. Bootstrap values above 50% are shown on nodes.

In our DNA barcoding tree (Figure 6) *Hy. pastanai* is recovered in a clade with three populations of *Hy. ericae* and *Hyphessobrycon ribeiroi*. This clade support is moderate (69%) and is recovered as sister group of *Hy. herbertaxelrodi* with low support. All these species are part of a clade with 72% bootstrap support that includes *Hyphessobrycon mamuruensis*, *Hy. montagi* and the peruvian species *Hy. nigricinctus* and *Hy. loretoensis*, and which is recovered as sister group of a clade with 96% support that includes mostly species of the putative *Hy. heterorhabdus* species group, but also with *Hemigrammus bellottii* and *Hy. eschwartzae*. All these species are recovered as the sister group of *Hy. agulha* and are part of the *Hy. agulha* lineage (in red, with 73% bootstrap support). Details of ASAP and Bayesian PTP (bPTP) species delimitation methods are provided in S1, and S2 results are equivalent, distinguishing all nominal species of the *Hy. agulha* lineage, including *Hy. pastanai*, but also separating *Hy. ericae* populations as three different species. Genetic distances are presented in Table 3.

**TABLE 3:** Genetic distances between *Hyphessobrycon pastanai* and the most similar species, estimated using DNA barcoding methodology.

	01	02	03	04	05	06	07	08	09	10	11	12	13	14	15	16	17
01‐*Hyphessobrycon pulchripinnis*																	
02‐*Hyphessobrycon agulha*	22.7%																
03‐*Hyphessobrycon cantoi*	23.3%	20.0%															
04‐*Hyphessobrycon heterorhabdus*	22.5%	19.3%	12.0%														
05‐*Hemigrammus bellottii*	22.0%	20.3%	12.6%	10.2%													
06‐*Hyphessobrycon amapaensis*	23.1%	18.8%	11.3%	11.0%	9.6%												
07‐*Hyphessobrycon Eschwartzae*	21.6%	18.6%	8.9%	9.3%	9.6%	8.7%											
08‐*Hyphessobrycon sateremawe*	21.4%	18.7%	10.1%	9.1%	8.4%	7.4%	7.1%										
09‐*Hyphessobrycon loretoensis*	21.2%	17.6%	17.4%	16.0%	17.1%	16.8%	17.8%	16.2%									
10‐*Hyphessobrycon nigricinctus*	20.3%	15.8%	15.9%	14.9%	14.1%	15.2%	12.6%	13.0%	9.3%								
11‐*Hyphessobrycon herbertaxelrodi*	22.1%	19.3%	14.9%	16.7%	15.1%	16.5%	15.6%	14.6%	12.8%	12.4%							
12‐*Hyphessobrycon ericae* Curuá Una	22.5%	17.8%	16.4%	15.9%	16.3%	16.8%	15.9%	14.7%	11.0%	10.3%	9.5%						
13‐*Hyphessobrycon ribeiroi*	21.6%	16.6%	16.2%	16.4%	16.3%	16.2%	16.6%	14.6%	11.8%	11.1%	11.0%	5.9%					
14‐*Hy. ericae* Porto Velho	21.6%	16.3%	16.6%	14.7%	14.2%	15.8%	13.7%	13.7%	12.1%	10.7%	9.6%	7.1%	7.2%				
15‐*Hy. ericae* Camaiu	21.7%	16.5%	16.0%	15.4%	14.4%	15.5%	15.5%	14.6%	12.3%	10.2%	9.2%	6.3%	5.1%	6.0%			
**16‐*Hyphessobrycon pastanai***	**22.3%**	**16.8%**	**17.0%**	**15.8%**	**14.8%**	**16.2%**	**15.6%**	**14.1%**	**12.9%**	**10.3%**	**9.5%**	**6.3%**	**5.7%**	**5.0%**	**4.3%**		
17‐*Hyphessobrycon montage*	21.6%	18.2%	17.7%	18.6%	18.3%	18.3%	17.7%	16.3%	12.8%	10.7%	13.5%	12.3%	11.7%	11.7%	11.0%	**10.7%**	
18‐*Hyphessobrycon mamuruensis*	21.7%	17.3%	14.5%	15.8%	16.8%	14.7%	15.2%	14.3%	11.9%	10.4%	11.6%	11.1%	10.1%	10.6%	9.5%	**9.3%**	9.3%

## 4 DISCUSSION

*Hyphessobrycon pastanai* is a member of the *Hy. agulha* lineage (sensu Faria et al., 2025), one of the four main clades of Hyphessobryconinae recovered in Melo et al. (2024) and defined as the least inclusive clade with *Hy. agulha* and *Hy. heterorhabdus*. Despite lacking a formal diagnosis, all confirmed species whose living colour pattern is documented have a thin midlateral iridescent stripe, and the majority have a dark longitudinal pattern, which can be a midlateral dark stripe or a broad diffuse dark stripe that occupies most of the ventral half of the body (Faria et al., 2025; Melo et al., 2024). Most of the species confirmed for this lineage were anteriorly assigned to the *Hy. heterorhabdus* and *Hy. agulha* species group (see introduction for species list and definitions of these species groups), with the only exceptions being *Hemigrammus bellottii* and *He. rubrostriatus*.

Our allocation of *Hy. pastanai* to the *Hy. agulha* lineage is based on the colour pattern similarity with the overall pattern of the lineage, the position in which the new species is recovered in our DNA barcoding tree, and the low genetic distance to *Hy. ericae*, a species confirmed as part of this lineage (Melo et al., 2024). *Hyphessobrycon pastanai* have both the iridescent and dark stripes typically seen in species of the *Hy. agulha* lineage, with the iridescent stripe being followed dorsally by a red stripe and ending in a red posterodorsal blotch (a condition also known in *He. bellottii*, *Hy. agulha*, *Hy. heterorhabdus*, *Hy. cantoi*, *Hy. ericae*, *Hy. montagi*, *Hy. mamuruensis* and *Hy. sateremawe*) and a dark longitudinal pattern represented by the thin midlateral dark stripe (which is very similar to the one observed in *Hy. agulha*). *Hyphessobrycon agulha* is the described species which presents higher superficial similarity with *Hy. pastanai* (compare Figures 1a and 4). This similarity is a consequence of both species presenting not only similar dark and iridescent stripes but also the presence of a red stripe running dorsally to the iridescent stripe and occupying the caudal peduncle as an elongated red blotch; very similar humeral blotches (i.e., horizontally elongated with ventral diffuse expansion on its anterior region); and the presence of caudal peduncle blotches (though the one of *Hy. agulha* is very faint, whereas the one of *Hy. pastanai* is blurred but conspicuous). Despite these similarities, our tree indicates that they are not closely related within the *Hy. agulha* lineage, with the molecular evidence suggesting that *Hy. pastanai* closest relatives, instead, are three populations of *Hy. ericae* from Rio Camaiu, Rio Curuá‐Una and Rio Madeira (which are indicated as different species by both the molecular species delimitation methods utilized) and *Hy. ribeiroi*.

*Hyphessobrycon pastanai*, *Hy. ericae* and *Hy. ribeiroi* form a clade with bootstrap support of 69% and possess relatively small genetic distances between them (varying from 4.3% to 6.3%, as seen in Table 3), especially when compared to the genetic distance between *Hy. agulha* and *Hy. pastanai* (16.8%). It is also noticeable that *Hy. ericae* is recovered in Melo et al. (2024) as sister species of *Hy. herbertaxelrodi* and deeply nested in the *Hy. agulha* lineage, whereas in our tree, both species are recovered as relatively closely related with the new species (with genetic distance of 9.2% between *Hy. pastanai* and *Hy. herbertaxelrodi* and 9.2%–9.6% between different genospecies of *Hy. ericae* and *Hy. herbertaxelrodi*).

*Hyphessobrycon ericae* is recovered as a species complex with similar genetic distances between each of the genospecies and *Hy. ribeiroi* and *Hy. pastanai*. Although this is reliable in indicating the species as closely related, the topology of the clade is unreliable due to the inability of COI to track precise phylogenetic relationships as it is a single gene. Despite that, we consider this as evidence of proximity of the clade's species and, more importantly, of the validity of *Hy. pastanai* as a new species.

The colour patterns of *Hy. pastanai, Hy. ribeiroi* and *Hy. ericae* are very distinct among them. Without considering colour patterns widespread in the *Hy. agulha* lineage (as iridescent stripes, dark stripes and reticulated dorsal half of body), these species share only the presence of caudal peduncle blotches and ventral diffuse expansions of the humeral blotch, and even these are observed in species seemingly not directly related with them (as *Hy. agulha*, for the humeral blotch ventral expansion and *Hy. montagi* and *Hy. mutabilis* for the caudal peduncle blotch). There are also conspicuous differences between their humeral and caudal peduncle blotches, with *Hy. ericae* and *Hy. pastanai* sharing the presence of a horizontally elongated humeral blotch, which differs in its posterior extension (*Hy. pastanai* possesses a humeral blotch that becomes diffuse in its posterior region, whereas the humeral blotch of *Hy. ericae* typically stays more well defined until disappearing), and *Hy. ribeiroi* having a mostly vertically elongated humeral blotch with a thin and short posterior expansion. The caudal peduncle blotches are very similar between *Hy. ribeiroi* and *Hy. ericae*, being approximately elliptic or rounded and tall enough to occupy most of the caudal peduncle height. *Hyphessobrycon pastanai* caudal peduncle blotch is very distinct from those by being narrower and restricted to the ventral half of the caudal peduncle (above ventral procurrent rays) and proximal region of the caudal‐fin middle rays and, in some individuals, ventral lobe.

The main exclusive similarity observed between *Hy. pastanai*, *Hy. ericae* and *Hy. ribeiroi* is the sexual dimorphism composed of bony hooks restricted to the pelvic fins (Lima et al., 2025; present study). This arrangement of bony hooks, though, has not been observed in all populations of *Hy. ericae*, being observed only in specimens from the Rio Camaiu basin (Lima et al., 2025). *Hyphessobrycon mamuruensis* (Faria et al., 2025) also have this bony hook distribution, but although this species is recovered as relatively close to *Hy. pastanai* (with a genetic distance of 9.2%), it is recovered not as part of the clade (*Hy. pastanai*, *Hy. ericae* and *Hy. ribeiroi*), but also as sister species of *Hy. montagi*, species in which no bony hook has been observed. It is still remarkable that this pattern is not known in any other species of the *Hy. agulha* lineage but the ones mentioned before, with species confirmed as part of the lineage typically having bony hooks on the pelvic and anal fin (present in *He. rubrostriatus*, *He. bellottii*, *Hy. agulha*, *Hy. amapaensis*, *Hy. cantoi*, *Hy. heterorhabdus*, *Hy. sateremawe* and *Hy. peruvianus*) or no bony hooks (as in *Hy. montagi*) (Faria et al., 2021; Faria et al., 2025; Faria, Bastos, et al., 2020; Lima et al., 2014).

Many phylogenetically untested species are highly similar with species confirmed as part of the *Hy. agulha* lineage and, therefore, likely to be part of it. Our tree indicates that *Hy. cantoi*, *Hy. eschwartzae*, *Hy. montagi*, *Hy. mamuruensis*, *Hy. nigricinctus* Zarske and Géry, 2004, and *Hy. loretoensis* are part of this lineage, all of them sharing both its dark and iridescent stripes, though with considerable variation in colour intensity and broadness. Species whose longitudinal dark stripes included them in the *Hy. agulha* or *Hy. heterorhabdus* species groups, as *Hyphessobrycon borealis*, *Hy. clavatus*, *Hy. klausanni, Hy. metae, Hy. lucenorum*, *Hy. zoe*, and *Hy. wosiackii* are also likely part of the same lineage due to the overall similarity with species already confirmed in the lineage. The most uncertain species among these is *Hy. lucenorum*, which, although exhibiting the diagnostic features of the *Hy. agulha* species group, lacks an iridescent stripe (as seen in fig. 5 of Ohara & Lima, 2015) and presents sexually dimorphic chromatism, features not shared by any of the confirmed species of the *Hy. agulha* lineage.

There are also *Hyphessobrycon* species that present strong similarity with species of the *Hy. agulha* lineage but were never included in any putative group. *Hyphessobrycon nigricinctus* would fit in this category but have been recovered in our present work as part of the *Hy. agulha* lineage, showing that midlateral stripes may be strongly marked, and not only entirely or partially diffuse (or blurred). Similar stripes are found in *Hyphessobrycon chiribiquete* García‐Alzate, Lima, Taphorn, Mojica, Urbano‐Bonilla & Teixeira 2020, and *Hyphessobrycon petricolus* Ohara, Lima & Barros, 2017, species with an overall similarity in colour pattern with *Hy. agulha* lineage (i.e., presence of a horizontally elongated humeral with a ventral diffuse expansion, which is followed posteriorly by a dark stripe and presence of an iridescent stripe that becomes red on the caudal peduncle, a condition that can be observed in all species of the *Hy. agulha* lineage but *Hy. herbertaxelrodi*), but with more strongly marked dark midlateral stripes. Therefore, we hypothesize that *Hy. petricolus* and *Hy. chiribiquete* are part of the *Hy. agulha* lineage, despite not being part of the *Hy. agulha* and *Hy. heterorhabdus* species groups.

Despite being part of Hyphessobryconinae, the *Hy. agulha* lineage is phylogenetically distant from the type species of *Hyphessobrycon*: *Hyphessobrycon compressus* (Melo et al., 2024). For the present moment, our molecular results and observed morphological similarities are enough to consider *Hy. pastanai* as part of the *Hy. agulha* lineage in Hyphessobryconinae, and both the genetic distance and differences towards species of this lineage corroborate its validity as a new species. However, more phylogenetic and morphological data must be gathered to support the description of supraspecific entities for the lineage.

Regarding its conservation status, four expeditions have been conducted in the Apuí region since 2016, during which *Hy. pastanai* was recorded only from its type locality, one stream next to the BR‐230 highway in Apuí, state of Amazonas, Brazil. This stream passes through a large tube beneath the road, followed by a vegetation‐cleared area with a small lake whose function is unknown to us. Despite being very close to the highway, therefore being at the border of a deforested area, there were trees protecting the stream from direct sunlight (and likely providing allochthonous resources to the stream community) up‐ and downstream of these anthropically impacted points. Although the type locality is considered as relatively preserved, which gives us little information about its resistance to anthropic impacts, it is located 8 km south from Floresta Nacional (Flona) do Aripuanã. This Flona also protects nearly 34 km of the main channel of Rio Jatuarana, and even upstream to this point, there seems to exist deforestation only around the BR‐230 road, with most of the area the river flows through being preserved forest according to satellite images. The distribution of *Hy. pastanai* likely exceeds the Rio Jatuarana basin, which means it potentially exists in other conservation units of the Apuí mosaic of federal protected lands created to diminish the regional deforestation rate. Therefore, we suggest the conservation status of *Hy. pastanai* as low concern (LC) according to the International Union for Conservation of Nature (IUCN) classification (IUCN Standards and Petitions Committee, 2024). However, Apuí has been among the regions most impacted by deforestation in recent times (Amorim et al., 2024; Amorim et al., 2025), and the Floresta Nacional do Jatuarana has figured among the Brazilian conservation units with more deforestation in a distance of less than 10 km from its limits between January and March 2022 (Amorim et al., 2022), despite being apparently nearly intact within its limits. It is also concerning that, during the government of Michel Temer, national news reported political pressure for the reduction in the Apuí mosaic through the downsizing of Reserva Biológica do Manicoré, Parque Nacional do Acari, Floresta Nacional do Aripuanã, Floresta Nacional do Urupadi and the degazettement of Área de Proteção Ambiental dos Campos de Manicoré (Farias & Pontes, 2017). More recently, during the Bolsonaro government, the environment ministry led by Ricardo Salles was reported as planning to review all 334 Brazilian conservation units, with the intention of downgrading and even revoking an uncertain number of them (Borges, 2019). These examples indicate that, although conservation units of Apuí were not altered until the writing of the present work, political pressures against conservation efforts in the region may induce reductions in environmental protection in Apuí, which together with the regional deforestation rates could become a threat, or at least a source of habitat loss, for *Hy. pastanai*.

## AUTHOR CONTRIBUTIONS

Tiago C. Faria: ideas, data generation, data analysis, manuscript preparation and funding (collection). Claudio Oliveira: data generation, data analysis, manuscript preparation and funding (collection and molecular data). Iann Leonardo Pinheiro Monteiro: data generation. Willian M. Ohara: data generation and funding (collection).

## FUNDING INFORMATION

Specimens of *Hyphessobrycon pastanai* were collected in two expeditions, the first partly funded by the project ‘South American Characiformes Inventory’ (FAPESP 2011/50282–7), and the second funded by the project ‘Filogenia e identificação molecular de peixes da superordem Ostariophysi (Chordata: Actinopterygii) utilizando abordagens genômicas’ (FAPESP grant 2020/13433‐6) and by the technical reserve of the first author PhD project ‘Sistemática e filogeografia dos tetras do complexo *Hyphessobrycon pulchripinnis* (Characiformes: Characidae)’ (FAPESP grant 2021/00242‐0).

The authors were funded by FAPESP (Willian M. Ohara, grant 2013/22473–8; Tiago C. Faria, grant 2021/00242‐0). Claudio Oliveira also received financial support from Conselho Nacional de Desenvolvimento Científico e Tecnológico – CNPq proc. 306054/2006‐0 and 441128/2020‐3, and Pro‐Reitoria de Pesquisa da Universidade Estadual Paulista Júlio de Mesquita Filho (Prope‐UNESP).

## Supporting information


**Data S1.** Assemble Species by Automatic Partitioning (ASAP) delimitation results.

**Data S2.** Poisson Tree Process (PTP) delimitation results.
