# Supplementary figures and images for: A new Hyphessobrycon (Characiformes: Acestrorhamphidae) of the Hyphessobrycon agulha lineage of Hyphessobryconinae from the lower Aripuanã basin, Brazil, with comments about the lineage
Source: J Fish Biol. 2026 Feb 26;109(1):58–69. doi: 10.1111/jfb.70379 (PMC13397144; doi:10.1111/jfb.70379)

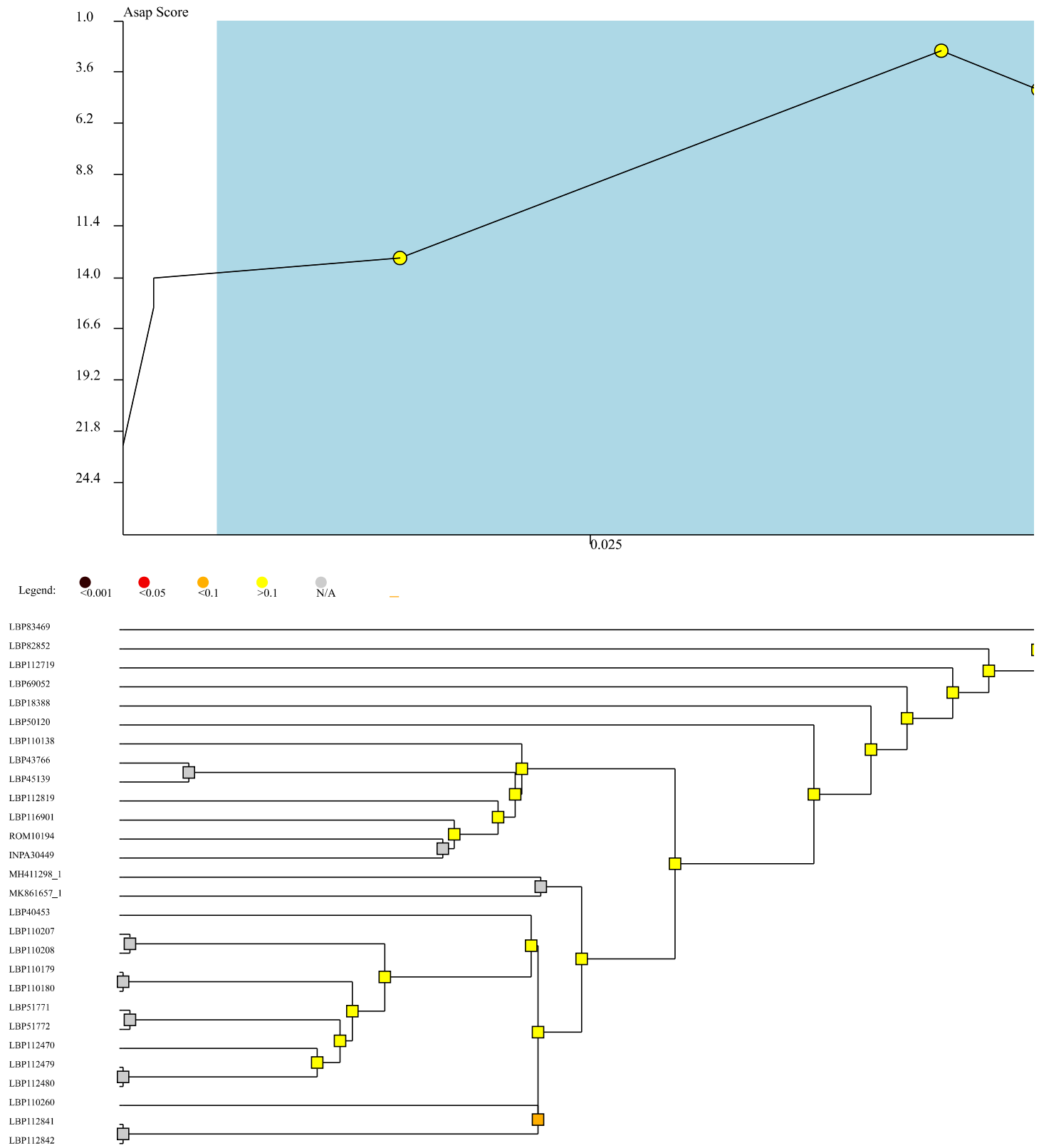

Supplement: Supplementary file 1 — Data S1. Assemble Species by Automatic Partitioning (ASAP) delimitation results. [file JFB-109-58-s001.pdf]
