# Supplementary material for: A new Hyphessobrycon (Characiformes: Acestrorhamphidae) of the Hyphessobrycon agulha lineage of Hyphessobryconinae from the lower Aripuanã basin, Brazil, with comments about the lineage
Source: J Fish Biol. 2026 Feb 26;109(1):58–69. doi: 10.1111/jfb.70379 (PMC13397144; doi:10.1111/jfb.70379)
Supplement: Supplementary file 2 — Data S2. Poisson Tree Process (PTP) delimitation results. [file JFB-109-58-s002.pdf]

BPTP SPECIES DELIMITATION

bPTP web server (/ptp/)

Look up jobs (/findjob/)

Help (/help/)

About PTP and bPTP (http://sco.h-its.org/exelixis/web/software/PTP/index.html)

PTP paper (http://bioinformatics.oxfordjournals.org/content/29/22/2869)

Download PTP and bPTP (https://github.com/zhangjiajie/PTP)

PTP species delimitation results

Maximum likelihood solution:

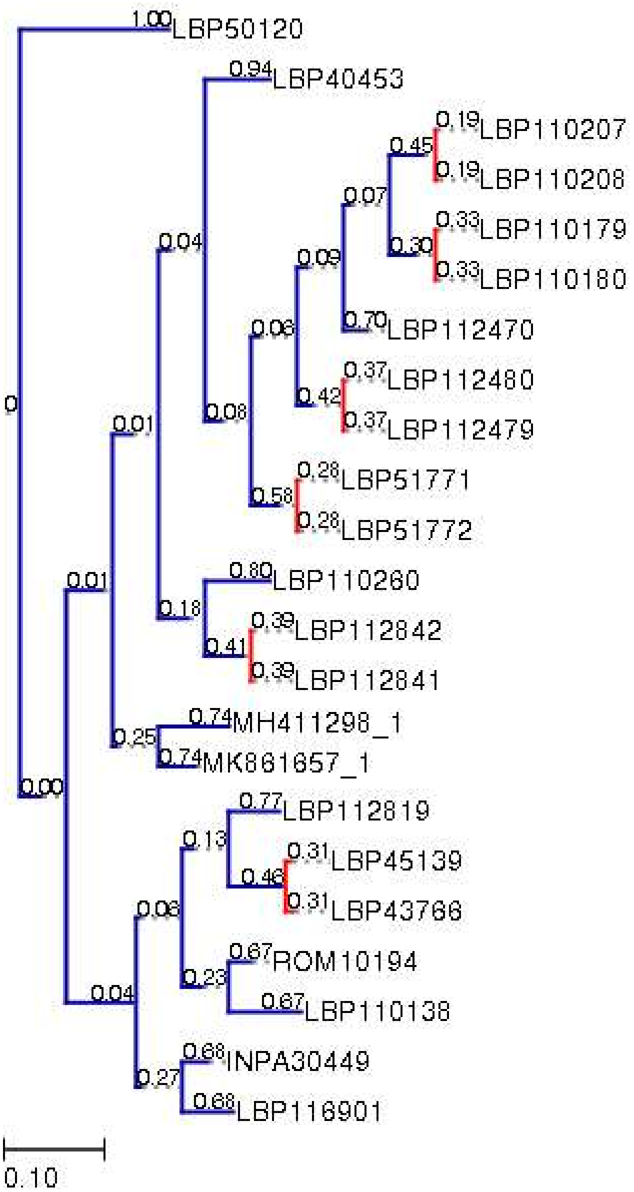

Download output in SVG (/download/3320/output.PTPMLPartition.txt.svg)

Download delimitation results (/download/3320/output.PTPMLPartition.txt)

Download annotated tree (/download/3320/output.PTPMLPartition.txt.ml.tre)

Highest Bayesian supported solution:

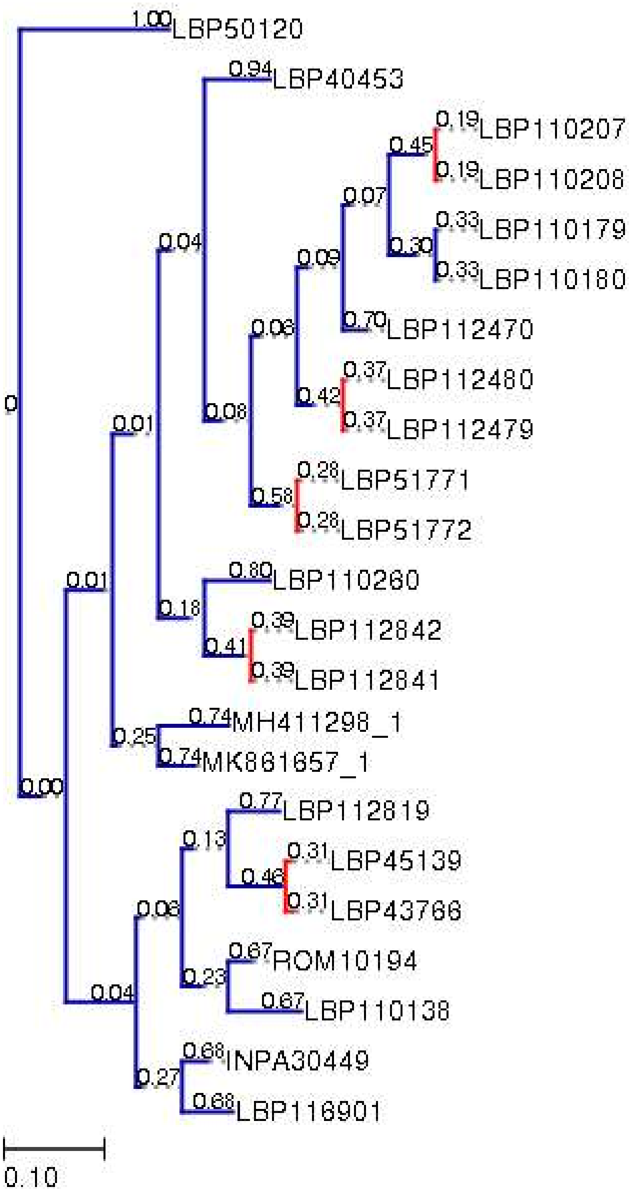

Download output in SVG (/download/3320/output.PTPhSupportPartition.txt.svg)

Download delimitation results (/download/3320/output.PTPhSupportPartition.txt)

Download annotated tree (/download/3320/output.PTPhSupportPartition.txt.sh.tre)

Download Bayesian results (/download/3320/output.PTPPartitions.txt)

Download likelihood trace file (/download/3320/output.PTPllh.txt)

Download likelihood trace plot in pdf (/download/3320/output.llh.pdf)

Download delimitation summary (/download/3320/output.PTPPartitonSummary.txt)

Accptance rate: 0.58423000000000003
Merge: 49910
Split: 50069
Estimated number of species is between 2 and 22
Mean: 16.30

PhyloMap-PTP

Show PhyloMap visualization of the maximum likelihood PTP species delimitation (/ptp/phylomap/?job_id=3320&email=tc.faria@gmail.com)

© Jiajie Zhang 2013-2015
Contact through google-group (https://groups.google.com/forum/?hl=en#!forum/ptp-species-delimitation)
